# Detecting adaptive convergent amino acid evolution

**DOI:** 10.1098/rstb.2018.0234

**Published:** 2019-06-03

**Authors:** Carine Rey, Vincent Lanore, Philippe Veber, Laurent Guéguen, Nicolas Lartillot, Marie Sémon, Bastien Boussau

**Affiliations:** 1 ENS de Lyon, CNRS UMR 5239, INSERM U1210, LBMC, Univ Lyon, Université Claude Bernard Lyon 1, F-69007 Lyon, France; 2 CNRS UMR 5558, LBBE, Univ Lyon, Université Claude Bernard Lyon 1, F-69100 Villeurbanne, France

**Keywords:** convergent evolution, genomics, molecular evolution, C3/C4, phylogenetics, probabilistic models

## Abstract

In evolutionary genomics, researchers have taken an interest in identifying substitutions that subtend convergent phenotypic adaptations. This is a difficult question that requires distinguishing *foreground* convergent substitutions that are involved in the convergent phenotype from *background* convergent substitutions. Those may be linked to other adaptations, may be neutral or may be the consequence of mutational biases. Furthermore, there is no generally accepted definition of convergent substitutions. Various methods that use different definitions have been proposed in the literature, resulting in different sets of candidate foreground convergent substitutions. In this article, we first describe the processes that can generate foreground convergent substitutions in coding sequences, separating adaptive from non-adaptive processes. Second, we review methods that have been proposed to detect foreground convergent substitutions in coding sequences and expose the assumptions that underlie them. Finally, we examine their power on simulations of convergent changes—including in the presence of a change in the efficacy of selection—and on empirical alignments.

This article is part of the theme issue ‘Convergent evolution in the genomics era: new insights and directions'.

## 1. Introduction

It is difficult to replicate experiments when we study evolutionary biology. However, one can benefit from natural replicates that have arisen through time and across taxa. Indeed, lineages that have adapted independently to a given environmental constraint can be seen as having been subjected independently to the same ‘experimental’ conditions. When lineages subjected to the same conditions evolve similar phenotypes, they are said to have *converged* in their phenotypes. In the rest of the article, we call ‘convergent lineages’ lineages that have undergone such convergent phenotypic evolution. In evolutionary genomics, researchers have taken an interest in identifying substitutions that subtend those convergent phenotypes.

We call these causative substitutions ‘foreground convergent substitutions'. We distinguish them from ‘background convergent substitutions’ that include substitutions that may be confused with ‘foreground convergent substitutions' but that have no phenotypic consequences on the studied convergent phenotype.

Foreground convergent substitutions may be adaptive, i.e*.* they fixed through positive selection, or non-adaptive, i.e*.* they fixed through a relaxation of selection (electronic supplementary material, figure S1). The latter may, for instance, occur in cases of regressive evolution, where a gene is no longer needed in a particular environment. Being able to distinguish these two types of convergent substitutions provides information about the underlying evolutionary process.

To identify foreground convergent substitutions, many methods search for substitutions that are correlated with the phenotype. These are substitutions that have occurred repeatedly in convergent lineages, towards the same derived state, or towards similar derived states (e.g. towards amino acids with similar biochemical profiles). Methods vary in how they quantify the similarity between substitutions, resulting in different sets of candidate substitutions [1–3]. Finding foreground convergent (causative) substitutions among many substitutions in genomes containing billions of sites is a challenge in modern bioinformatics.

Several processes at work in genome evolution may affect the number of convergent substitutions in a given dataset. For instance, mutational biases, changes in recombination rates, biased gene conversion (bGC) or changes in population size may inflate or diminish the number of convergent substitutions. They may also affect differently the numbers of foreground and background convergent substitutions. Although this has not been studied yet, one may assume that these complex processes make it harder for methods to distinguish foreground from background convergent substitutions.

In this article, we first describe some processes contributing adaptive and non-adaptive foreground convergent substitutions in coding sequences. Second, we review existing methods to detect foreground convergent amino acid substitutions and expose the assumptions that underlie them. Third, we examine their power on simulations of convergent changes—including in the presence of variations in selection efficacy—and on two empirical alignments.

### (a) Defining adaptive convergent amino acid evolution

In this section, we examine how foreground convergent substitutions can arise through adaptive processes. To this end, it is useful to first discuss adaptive genomic evolution in general. Adaptive genomic evolution is expected to occur when constraints on the phenotype change, which alters the selective pressure at some sites in the genome. Individuals with mutations that provide an increased fitness in the new environment have a reproductive advantage. Such mutations then increase in frequency and can eventually fix. The fixation of one or more of these mutations can, in turn, change the selective pressure on other sites of the genome through epistatic interactions [4].

The characteristics of the *fitness landscape* have an impact on how likely adaptive convergent evolution is. The fitness landscape describes the mapping between genotypes and fitness in a species, for a given set of constraints on the phenotype. Because it treats the genotype as a whole, it naturally considers all the sites of the genome and their interactions at once. If it is highly peaked, it means that only one genotype can provide the largest fitness. In that case, one can expect that several related species under the same constraints on their phenotype may adaptively converge towards the same genotype, i.e*.* adaptive convergent substitutions are likely. Instead, if the fitness landscape is very flat, different genotypes can provide similar fitnesses, so that several related species under the same constraints on their phenotype may move towards different genotypes, making adaptive convergent substitutions less likely.

These intuitive considerations should make it clear that a good mechanistic model of convergent evolution needs to consider the entire genome at the same time, along with the fitness landscape, to take into account all the dependencies between sites. For computational reasons, and because fitness landscapes are only rarely studied experimentally [4], such a model is currently out of reach. Instead, sites share some general parameters ruling their evolution (e.g. branch lengths, some parameters of the substitution matrices) but, conditionally on those parameters, each site is typically modelled independently of the others. In addition, many models make simplifying assumptions; for instance, fitness landscapes only depend on the phenotype, and not on the lineage under consideration. All models that have been developed to detect convergent genomic evolution assume such site-independent models.

**Figure 1.**
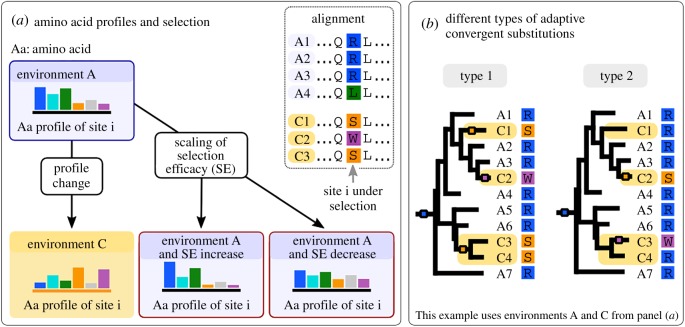
Categories of adaptive and non-adaptive convergent amino acid evolution. (*a*) At a particular position in a protein, some amino acids provide better fitness than others. This is represented by coloured bars for six amino acids, the bigger the bar the higher the fitness. In the ancestral environment A, amino acids blue and green provide the highest fitness, whereas in the convergent environment C, amino acids orange and purple provide the highest fitness. Increasing the selection efficacy makes the profiles more pointed, while decreasing it makes them more flat, but the amino acid relative rank does not change. Decreases of the selection efficacy are not adaptive, while the two other types of changes are. (*b*) Species with the convergent phenotype are named C* and species with the ancestral phenotype are named A*. Substitutions are represented by small boxes on the branches. We distinguish two types of adaptive convergent substitutions. Type 1 are substitutions that occur systematically on the branch where the phenotype changes, at the transition between Ancestral and Convergent environments (A–C). Type 2 are substitutions that occur on later branches (e.g. in the branch leading to C3).

In this article, we propose to define convergent evolution through the comparison of coding sequences across species. Coding sequences offer a window into where the mutation process and the selective process meet. Indeed, non-synonymous mutations (i.e. mutations that change the encoded amino acid) might be strongly selected, while synonymous mutations (i.e. mutations that do not change the encoded amino acid) should be neutral or weakly counter-selected. Natural models to study coding sequences are codon models, in which one site is made of three nucleotides encoding a particular amino acid. The simplest codon models consider one site at a time, independently of other sites, and distinguish between synonymous and non-synonymous substitutions. They assume that synonymous substitutions provide a proxy for the rate of fixation of neutral substitutions, while all non-synonymous substitutions have the same rate of fixation, which depends on selection efficacy [5,6]. More sophisticated codon models distinguish between different amino acid changing substitutions and assume that different amino acids provide different fitnesses. Such models use amino acid fitness profiles—which we simply call *amino acid profiles* in the rest of the article (figure 1) [7]. Some of the richest models allow individual fitness profiles for different sites [8,9]. Overall, codon models provide a convenient framework to define adaptive convergent amino acid evolution.

In a simple model that considers one codon at a time, adaptive convergent evolution can result from an increase in the selective pressure or from a change in its nature. Increases in the pressure would mean that, in the amino acid profile at a given codon, the amino acids that provided high fitness before the environmental change provide even higher fitness, while amino acids providing low fitness before the change now provide even lower fitness (figure 1*a*, ‘Scaling of Selection Efficacy’). It has become more important for the organism to have particular amino acids at this position. For example, this could be associated with a lifestyle where the function of the protein has become more important than it was. Changes in the nature of the selective pressure manifest themselves by a change between two amino acid fitness profiles, referred to as ‘ancestral’ and ‘convergent’ from now on (figure 1*a* ‘profile change’). As opposed to increases in the pressure, profile changes alter which amino acids are the most fit at a given position.

In the rest of the article, we distinguish between two types of adaptive convergent substitutions (figure 1*b*), because detection methods vary in their ability to detect each type. We call ‘type 1 substitutions' the early substitutions that occur on the branch where the phenotype changed, and ‘type 2 substitutions’ those that fix after type 1 substitutions, on subsequent branches.

### (b) Non-adaptive background convergent amino acid evolution

Background convergent amino acid substitutions may be linked to convergent phenotypes that have not been detected, or not linked to convergent phenotypes, and possibly have no phenotypic consequences. In this latter case, they arise non-adaptively. Some number of such non-adaptive background convergent substitutions is expected, if only because there are only 20 possible amino acids. Further, the structure of the genetic code and the characteristics of the mutation process (e.g. that transitions are more frequent than transversions) all contribute to making some amino acid substitutions more likely than others and therefore increase the probability that they will be convergent.

In addition, fixation and mutation biases could create patterns resembling adaptive convergent evolution, and possibly adaptive foreground convergent evolution. In particular, GC-bGC is a fixation bias that favours G or C alleles over A or T alleles and is widespread across the tree of life [10,11], and CpG hypermutability is a well-known mutation bias. bGC is most intense in regions of the genome that recombine frequently and has a stronger effect over time in species with large effective population sizes and short generation times. Those two characteristics have appeared independently several times in the tree of life. Because of bGC, one can expect to detect similar changes to GC alleles in the species sharing these characteristics, even without any adaptive value to having GC alleles instead of AT at those positions. This phenomenon seems to be strong enough to affect single gene phylogenies in birds [12,13] and may be an important driver of background convergent sequence evolution. CpG hypermutability results from a higher rate of mutations of methylated CG dinucleotides and could also contribute to background convergent sequence evolution. It has also been shown to promote foreground convergent evolution, with recurrent changes at the same CpG site in passerine bird haemoglobin [14].

Repeated and independent global relaxations of selection could also create background convergent evolution. If the phenotypic change is linked to a genome-wide decrease in selection efficacy, e.g. through a decrease in the effective population size [15], mutations that used to be counter-selected become tolerated. Because of the structure of the genetic code, those mutations could result in similar amino acid substitutions in lineages undergoing the decrease in selection efficacy.

Finally, epistatic interactions between sites in the genome or within a protein can create non-adaptive convergent amino acid evolution [16,17]. The same mutation at a particular site can occur in independent lineages simply because by chance sites that are in epistatic interactions with it happen to be in the same state in those lineages. The mutation therefore fixes not because of an adaptation to a new environment, but because of the states of interacting sites. Such non-adaptive convergent evolution is more likely in closely related lineages than in distant lineages [16]. It is difficult to know how frequent such events are, in part because most of the models used to study protein evolution have ignored epistatic interactions. We do not study such phenomena in this article but acknowledge that they may be an important confounding process in the search for adaptive convergent amino acid evolution.

### (c) Detecting adaptive foreground convergent amino acid substitutions

**Figure 2.**
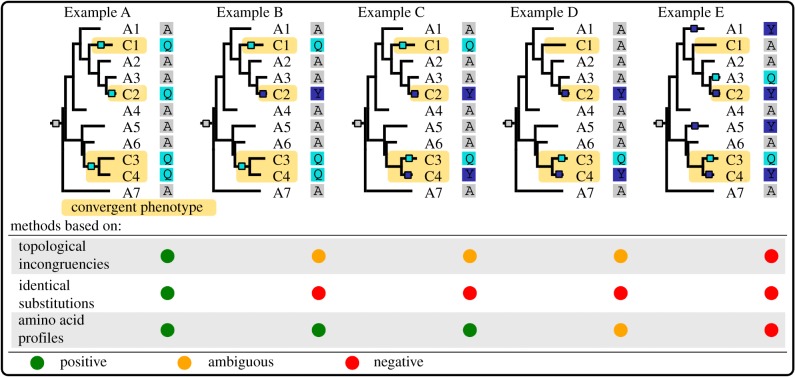
Cartoon examples of the types of sites targeted by each type of method. The tree topologies and species are the same in all examples. Species with the convergent phenotype are named C*, those with the ancestral phenotype A*; the transitions between ancestral and convergent phenotype occur where the subtrees become shaded in yellow. Coloured squares on the branches of the phylogeny indicate substitution events, with the colour corresponding to the new amino acid. In Example A, every time the phenotype changes, a substitution occurs towards amino acid Q (type 1 substitutions to a single amino acid). This is an ideal case for the methods based on identical substitutions and should be detectable by all methods. Example B shows a site that has undergone a profile change, whereby two different amino acids, Q and Y, have good fitness in the convergent case. All methods but the identical may detect such changes, although this depends on how different the ancestral and the convergent profiles are [18]. Example C is similar to Example B except that some substitutions occurred after the phenotype has changed (type 2 substitutions), not simultaneously with the phenotype change. Example D is similar to Example C except that the amino acid change only occurred three times out of four: this makes it more controversial and harder to detect. But if the change in profile is strong enough, profile methods should be able to detect it. Example E shows a case where the evolution of the site does not seem to correlate with the convergent/ancestral state of the species. We do not expect the methods to detect such a site, but some such sites will nevertheless come out as false positives.

Several methods have been designed to detect adaptive convergent amino acid evolution. We list them below and attempt to predict their relative strengths and weaknesses, in particular, their capacity to detect type 1 and type 2 adaptive convergent substitutions. Figure 2 presents in cartoon format the type of convergent sites that each type of method should be able to detect. None of the following methods have been designed to detect convergent increases or decreases in selection efficacy, so we expect they should do much better at detecting convergent profile changes than convergent changes in selection efficacy. All methods except one (msd, [19]) assume that convergent lineages are known without uncertainty and that corresponding clades are given as input.

#### (i) Methods looking for independent substitutions to the same amino acid

The most intuitive method, the ‘identical’ method, looks for independent substitutions to the exact same amino acid in all clades with the convergent phenotype [20,21]. It therefore assumes that a particular amino acid has a much better fitness than all other amino acids at this particular position of a protein. In practice, it relies on ancestral sequence reconstruction to infer the amino acids present before each convergent transition and make sure that the transition of interest occurred on the branch where the phenotypic transition occurred. By design, it is very conservative because it aims to detect only sites where a single particular amino acid is much more fit than others, which fixed with a type 1 substitution (figure 2).

An extension of this method, the ‘expectation’ method of Chabrol *et al.* [19]—also called msd—looks for sites with a high ‘convergence index’. This convergence index is the expected number of substitutions to a particular amino acid in convergent lineages. Interestingly, and contrary to the other methods presented here, this method does not assume that convergent lineages must be known. Instead, it is enough to have phenotypic annotations for extant species only. It is unclear whether this method is very conservative or not: on one hand, it detects only sites where a particular amino acid is found in most species with the convergent phenotype (as in the ‘identical’ method), but on the other hand, this convergence could apply to only a subset of the species with the convergent phenotype, an advantage compared to methods based on amino acid profiles (see below, §1c(iii)). Both type 1 and type 2 substitutions can be detected by this method, but type 2 substitutions get a higher convergence index than type 1 substitutions and may therefore be better detected.

#### (ii) Method based on topological incongruencies

The ‘topological’ method is an early attempt to look for an indirect effect of convergent sequence evolution, based on an observation first made on the prestin gene [22] and later systematized in genome-scale studies [1–3]. When a particular site has evolved convergently in several lineages, it displays the same or similar amino acids in those lineages, and not in lineages with a different phenotype. As a result, for this site, a phylogeny in which convergent lineages are grouped together is more likely than the true species phylogeny. This approach involves constructing the species topology and a ‘convergent’ topology where species with the convergent phenotype are grouped together. Then, each site can be tested for which topology it prefers—the true species phylogeny or the convergent phylogeny—by comparing the likelihoods of the two trees for this site. This method is capturing a byproduct of convergent evolution, and not its mechanism, hence it is difficult to know precisely what type of substitution this method can work with. Presumably, both type 1 and type 2 substitutions can be detected.

#### (iii) Methods based on amino acid profiles

‘Profile methods’ are methods aiming to detect selection pressure changes, whereby different amino acids provide the highest fitnesses in the ancestral and convergent phenotypes. The simplest of these methods is the ‘multinomial’ approach, which performs a simple *χ*^2^ test for multinomial distributions [23] between two vectors of amino acid frequencies. One vector is based on the amino acids found in extant species with the ancestral phenotype, and the other vector is based on the amino acids found in extant species with the convergent phenotype. This approach has not previously been used in the literature to our knowledge and suffers from a major drawback in that it fails to account for the phylogenetic structure of the data. However, we chose to include it in our tests as it provides a baseline against which the other more sophisticated methods can be tested. Both type 1 and type 2 substitutions can be detected by this method.

Other profile methods include profile change with one change (PCOC) [18], diffsel [24] and TDG09 [25], which belong to a family that we loosely call ‘mechanistic methods’ because they combine a phylogenetic approach with amino acid fitness profiles.

The ‘PCOC’ method [18] models convergent evolution at the amino acid level, without taking into account the codon level. It combines the ‘profile’ idea—by attributing to the 20 amino acids different equilibrium frequencies before and after the phenotypic changes—with the One Change (OC) model. OC assumes that convergent sites must have undergone a substitution on the branches where the adaptation took place. Detection of convergent sites is obtained by comparing the likelihoods of two nested models. In the first model, both the profile change and OC models are used—this means that profiles change on branches where the phenotype changes and that at least one substitution must occur on each of these branches. In the second model, evolution is homogeneous across all branches. Amino acid profiles are not estimated but are drawn from pre-existing distributions that have been estimated on large collections of alignments [26]. Both type 1 and type 2 substitutions can be detected by PCOC, but with a different power: the OC component of PCOC expects only type 1 substitutions, but the PC component can accommodate both type 1 and type 2 substitutions.

The TDG09 model [25] is similar to PCOC in that it works at the amino acid level, but it focuses on profile changes and does not include the OC component. In addition, it estimates the profiles separately for each site of the alignment. To do so, it builds two profiles, one for the species with the ancestral phenotype, and one for the species with the convergent phenotype. Amino acids with a count of 1 or less are considered absent, and all absent amino acids are assigned a 0.0 frequency in the profile vector. To detect sites undergoing adaptive convergent evolution, a likelihood ratio test is performed between a model that assumes a single profile across the entire tree, or two profiles for the ancestral and convergent parts of the tree. Both type 1 and type 2 substitutions can be detected by this method.

Finally, diffsel [24] is similar in spirit to TDG09 but works at the codon level and uses an MCMC algorithm to perform inference in the Bayesian framework. In this codon model, mutations occur at the DNA level, and selection occurs at the amino acid level. Selection is modelled as site-wise fitness profiles of 20 amino acid fitnesses. Convergent sites are characterized by a systematic change from an ancestral amino acid fitness profile to a different amino acid profile on all branches where the phenotype changed. Both type 1 and type 2 substitutions can be detected by this method.

## 2. Results and discussion

Some of the methods presented above have been implemented in several software packages (table 1). In this article, we evaluate these software packages on simulated and empirical data along with methods we have reimplemented ourselves. Regarding empirical data, we focus on sites that had been identified as convergent in previous publications and look at how the methods rank those sites. Regarding simulations, we evaluate the power of the methods in three cases: (1) a convergent profile change; (2) a convergent increase or decrease in selection efficacy; and (3) a combination of the above two, whereby a convergent profile change occurs simultaneously with a scaling of selection efficacy. To achieve this scaling, we set a selection efficacy parameter that is the product of two parameters, the population size (Ne) and the selective pressure (S) (also called scaled selection coefficient). In the following, we refer to this value by NeS, a composite parameter whose variations can be interpreted as e.g. a genome-wide variation of population size, or a site-wise variation of selective pressure. We choose to use NeS = 4 as the reference value, because it produces alignments similar to empirical alignments according to a range of statistics (electronic supplementary material, figures S4–S7).

**Table 1.** Summary of the methods used in the pipeline.

name	original method publication	level	executable or source available	median computing time on 2000 sites
identical	[20]	site	no, reimplemented (Python and C++)	55 s
topological	[1,27]	site	no, reimplemented (Python and C++)	5 s
TDG09	[19,25]	site	yes. Used a modified version. https://github.com/tamuri/tdg09	1648 s (27 min)
diffsel	[24]	site	yes: https://github.com/vlanore/diffsel	141084 s (39 h)
PCOC	[18]	site	yes: https://github.com/CarineRey/pcoc	181 s (3 min)
multinomial	—	site	no, implemented de novo (Python)	21 s
msd	[19]	gene	yes. Used a modified version. https://github.com/gilles-didier/Convergence	70 s

We ran the methods on four empirical phylogenies with different size, depth and number of transitions [20,28,29] (electronic supplementary material, figure S2).

**Figure 3.**
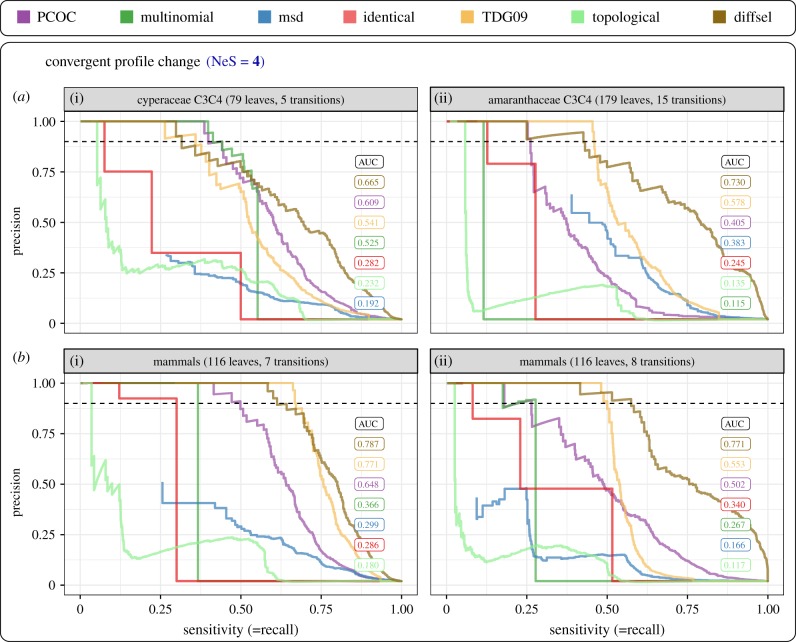
Detection of sites undergoing convergent profile change by different methods. Simulations are performed with constant selection efficacy (NeS = 4). Each panel corresponds to one empirical phylogeny, with convergent transitions placed as in electronic supplementary material, figure S2. The trade-off between sensitivity and precision is presented for each method, assuming that 2% of the sites are convergent in the sequences (colour code indicated on the top of the figure). The dashed lines highlight 90% precision. Area under the curves (AUC) ranked from best to worst are presented on the right-hand sides of each panel, with the same colour code as the precision-recall curves.

**Figure 4.**
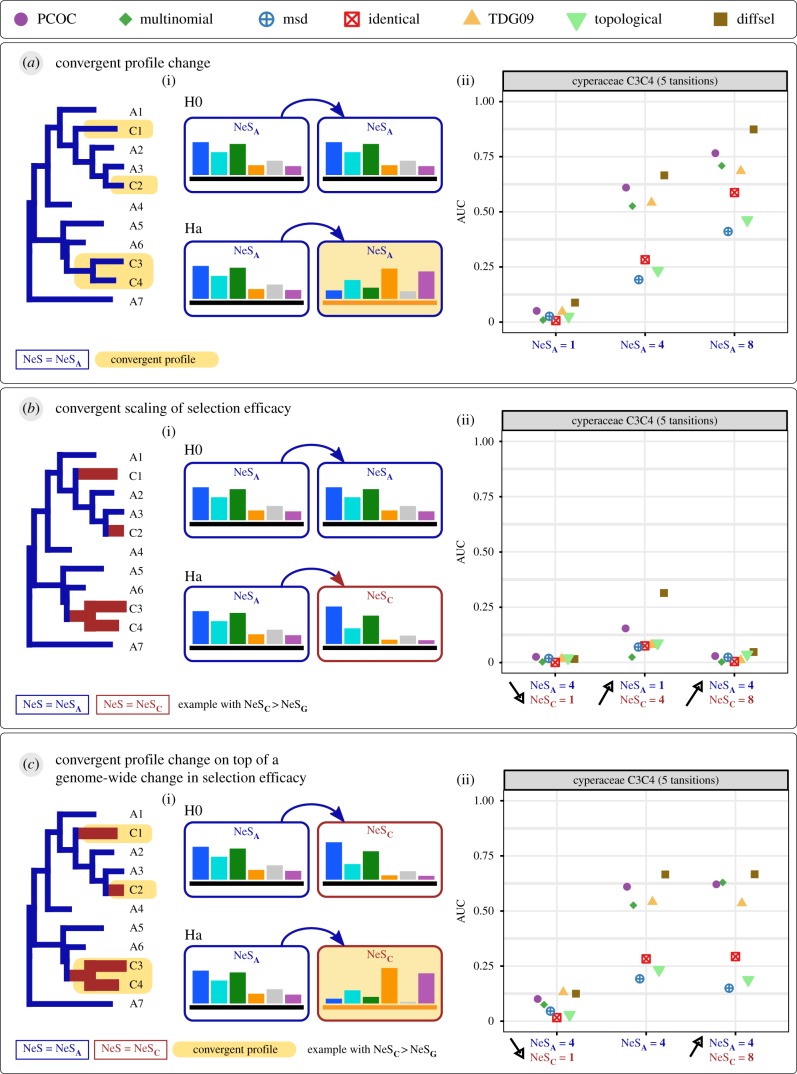
Overview of the simulations and AUC values for the Cyperaceae tree. The trees, convergent clades and symbols are as in figure 1. Three kinds of adaptive convergent cases have been simulated: (*a*) a convergent profile change, (*b*) a convergent scaling of selection efficacy and (*c*) a convergent profile change combined with a selection efficacy scaling. The genome-wide selection efficacy (NeS**_A_**) remains the same in (*a*) and is changed to a convergent selection efficacy (NeS**_C_**) in Ha (*b*) and Ha (*c*). The black arrows (*b*(ii) and *c*(ii)) indicate if selection efficacy increases or decreases in convergent clades. AUC values are calculated based on precision-recall curves such as presented in figure 3 ((*a*) AUC values for NeS_**A**_ = 4 in case 1 correspond to figure 3*a*(i)).

In case 1, ‘Convergent profile change’, selection efficacy remains constant but the amino acid profile is different in convergent lineages compared to the rest of the tree. To simulate this case (figure 3*a* and figure 4*a*), we change the amino acid profile in the convergent clades and we keep the same global NeS along the tree. The results are presented in figure 3 for NeS = 4, and for the four empirical phylogenies.

Profile methods perform better than the other methods in the four phylogenies, and among them, diffsel dominates the benchmark according to AUC values (figure 3). The sensitivity at 90% precision is not as easily interpretable as AUC because the curves are very rugged; TDG09, PCOC and diffsel seem to dominate this metric, with a different order depending on the tree. Surprisingly, the simplistic multinomial method performs well on the Cyperaceae tree, competing with the TDG09 and PCOC in terms of its sensitivity at 90% precision. The relative ranks of PCOC, multinomial and TDG09 vary depending on the tree, which may be attributable to differences in the number of convergent transitions and in the relative size of the convergent clades. For instance, we suspect that PCOC's performance is degraded when the number of convergent transitions increases, because by design it looks for sites with convergent changes in all the convergent clades, not just a subset of them. TDG09 shows the opposite trend, with better performance when the number of transitions increases. The topological, identical and msd approaches typically perform worse, but the AUC rank of msd is volatile. The low sensitivity of identical and msd is expected as those methods can only detect convergent substitutions to a particular amino acid, not to an amino acid profile. Overall, these results are qualitatively congruent with previously published simulations obtained with simpler settings and fewer methods [18]. However, the precisions and sensitivities observed here are much worse than those reported in [18], because simulations do not use the PCOC model, which enforces substitutions on all transition branches.

Note that diffsel, which performs well in our experiments, is also the most expensive method computationally by several orders of magnitude (table 1). Other methods may be preferable for large datasets unless extensive computing resources are available. The better performance of profile methods may be owing to their fitting the simulation conditions better. However, it is unclear how we could have simulated convergent evolution realistically without using mutation-selection models that use profiles of amino acid frequencies. In the end, this indicates that profile methods may perform better on empirical data as well; apart from diffsel, which always comes out first, the variability of the AUC ranks among trees, however, indicates that using several methods on a dataset is recommended.

We then studied the performance of the methods for a wider range of genome-wide selection efficacies, focusing on the Cyperaceae tree (see electronic supplementary material, figure S8 for the three other trees). Figure 4*a* represents AUC values for the Cyperaceae tree, for NeS = 1, 4 and 8, corresponding to values for weak, medium and high selection efficacy respectively, all of which produce alignments with realistic properties (electronic supplementary material, figures S4–S7). As expected, the methods are most accurate when NeS is high (NeS = 8) and the performance collapses when selection is not efficient (NeS = 1). In other words, it should be extremely difficult to detect convergent molecular evolution in species with small Ne, or for sites under weak selective pressure.

In case 2, ‘Convergent scaling of selection efficacy’ (figure 4*b*), the same amino acid fitness profile is used along the whole tree for a given site, but NeS is changed in convergent clades (from NeS**_A_** to NeS**_C_**) in Ha simulations. It is important to note that an NeS variation implies the modification of the fitness of each amino acid in the profile but not of its rank (figure 1*a*). We made 3 runs, two with an increase and another with a decrease of NeS in convergent clades. Overall, methods perform poorly at detecting selection efficacy scaling, with the exception of the NeS**_A_** = 1 to NeS**_C_** = 4 cases where PCOC and diffsel detect a small number of sites.

By the two previous cases, we saw that methods can detect adaptive convergent sites under two conditions: they have undergone a profile change and they are under moderate to high selective pressure. But the methods cannot detect profile changes when selection efficacy is low and also fail to detect scalings in selection efficacy alone.

Finally, case 3 introduces a confounding factor. Here we assume a genome-wide scaling of selection efficacy on top of which convergent sites undergo profile changes (figure 4*c*), and we try to detect those latter sites. This is modelled by a selection efficacy scaling from NeS**_G_** to NeS**_C_** in both convergent (Ha) and non-convergent (H0) sites, plus an amino acid profile change in Ha. We tried both to decrease (figure 4*c*(i)) or increase (figure 4*c*(ii)) the selection efficacy in the convergent clades and compared the results to the situation obtained when selection efficacy is constant. With a decreased selection efficacy in convergent clades, the methods' performances deteriorate compared to the reference simulation. With an increased selection efficacy in convergent clades, the performances remain roughly the same. In other words, a decrease in selection efficacy (for instance, owing to a decrease in Ne) coinciding with convergent transitions has a negative impact on the detection of convergent profile changes, but an increase has very little impact.

**Figure 5.**
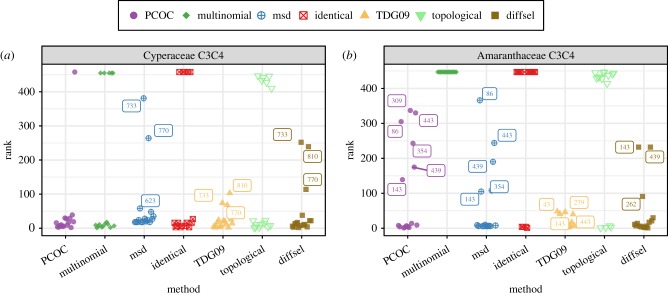
Ability of the different methods to recover published convergent sites in two empirical alignments. Those alignments had been used to study convergent transitions from C3 to C4 metabolisms in plants ((*a*) Besnard *et al.* [20] found 16 convergent sites in Cyperaceae, (*b*) Parto & Lartillot [24] found 15 convergent sites in Amaranthaceae). For each method, the scores were obtained for each site of the alignment. The sites were then ranked according to their scores, and only the ranks of previously published convergent sites are reported on the figure.

We then ran the seven methods on two previously published datasets, where a list of convergent sites had been proposed [20,24,28]. In these articles, the detection of convergent sites was performed by a version of the identical method for Cyperaceae, and by either diffsel or a dN/dS analysis for Amaranthaceae. Note that in the original diffsel article, the method was run with slightly different settings: it evaluated fitness profiles separately for the sister clades of convergent clades. In this article, diffsel was instead set up to only evaluate one profile per site for convergent clades and one profile per site for the rest of the tree. This change was done to make diffsel results more comparable with other methods and explains the differences with the original results. We compared the ranks of these previously reported convergent sites across methods. The alignments and the ranks are available in electronic supplementary material, figures S10 and S11, along with further discussion. Overall, the methods tend to agree with each other and rank the previously reported convergent sites among their best candidates (figure 5). In particular, most profile methods are in strong agreement with the publications; this is especially true for TDG09. Some methods fail to find any or nearly any convergent evolution on the Amaranthaceae alignment (identical, multinomial, topological), which is consistent with our results on data simulated on the same tree (figure 3*a*(ii)). These methods have a more consistent behaviour on the Cyperaceae dataset. For both datasets, most of the sites that have low ranks have low ranks across methods. For instance, this is the case for sites 733 and 770 in Cyperaceae, and 143 and 439 in Amaranthaceae. Overall, methods that produce the best results on simulated data also recover convergent sites identified in previous studies. Those sites had been identified with either diffsel or identical, so it is not surprising that these methods performed well in our study on the alignment on which they had been used; nonetheless, the general agreement between the methods is reassuring.

## 3. Material and methods

### (a) Simulation of alignments of coding sequences

We simulated coding sequences using bppseqgen [30] under (heterogeneous) mutation-selection models, which belong to the ‘mechanistic’ family of methods tested in this work. Mutation-selection models are codon models that combine mutations at the DNA level with amino acid fitness vectors, so that selection operates only at the amino acid level. Our mutation-selection models were complemented by a parameter indicating the efficacy of selection, NeS. In our mutation-selection model, NeS controls the flatness of the amino acid profiles (electronic supplementary material, §2). With a high NeS, the profiles are very peaked, and with a low NeS, very flat. We investigated the impact of different NeS values, in homogeneous models, where the same NeS is applied to all the branches (figure 4*a*), and in heterogeneous models, where different NeS are used for the branches in the ancestral and convergent parts of the tree.

We performed several types of simulations. Simulation settings are described in the results section (figure 4). For each simulated codon position, one or two profiles are selected randomly in our set of 263 non-redundant profiles and one or two NeS values are chosen. One profile and one NeS value are used for the ancestral branches, and the others for convergent subtrees.

### (b) Methods to detect foreground adaptive convergent substitutions

In order to compare results across methods, it was necessary to standardize their output. See the electronic supplementary material for details.

### (c) Pipeline and implementation of the methods

The results in this article were obtained using an all-in-one pipeline that encompasses simulations, detection and post-simulation analysis, including the generation of the plots used for figures 3 and 4. The pipeline itself was implemented in OCaml using bistro (https://github.com/pveber/bistro), a library to build statically typed reproducible workflows. Special attention was paid to reproducibility, in particular, by following the guidelines given in [31]. Instructions to reproduce our results are given in the electronic supplementary material.

The implementations of the methods used in the pipeline are as follows:

— The multinomial method has been implemented *de novo* in Python as well as the identical and topological methods which additionally use executables from the bppsuite [30]. They are available via the pipeline.
— The TDG09 implementation we used is a slightly modified version of the one available on github (table 1) where multithreading has been removed to avoid multithreading-related problems. Results should be identical to the github version. In addition, a script available in the pipeline repository was written to adapt input alignments and trees to TDG09 expected formats.
— For diffsel, we used an optimized version of the original implementation that is faster but implements the same model. The implementation we used is available on github (table 1). In addition, we use a different approach to establish MCMC convergence. The original method compares two MCMC chains using the tracecomp program from the PhyloBayes suite [32]. Instead, we run only one chain, use the Raftery and Lewis's Diagnostic implemented in the R package coda (v0.19-1) [33] after 200 iterations to estimate the number of necessary iterations, then run as many iterations as 120% of the estimated number and finally perform the same diagnostic to check convergence.
— We used the github version of PCOC (table 1) as is.
— Regarding msd, we used a version modified by the author so as to output a *p*-value for all sites, which we needed to compute scores.

The experiments performed for this article—i.e*.* the whole pipeline with 2000 sites for each hypothesis times 12 hypotheses times four trees—took 5 days to run on a 24-core virtual machine. Computation times observed during this run for individual detection methods are given in table 1. Note that most of the computing time for the whole pipeline is spent in diffsel tasks, which are a lot more costly to compute than other methods.

### (d) Using the methods on real alignments

We ran the methods on two previously published alignments: the Amaranthaceae alignment (447 sites, 15 published convergent sites) [24,28] and the Cyperaceae alignment (458 sites, 16 published convergent sites) [20]. The sites displayed in figure 5 are the sites proposed as possibly convergent in the original publications. Scores were obtained for each method and the sites were ranked (tied elements get the highest rank).

## 4. Conclusion

Our simulation results reveal the performance of existing methods to detect two different types of convergent amino acid evolution on simulated data, in isolation or combined with each other. The simulations have been performed with complex models of sequence evolution, parametrized so as to generate datasets that resemble empirical data on a few test statistics. However, some key assumptions underlying those models are clearly unrealistic: first, each site is simulated independently of the others. It would be useful to incorporate epistatic constraints in our simulations as those increase the number of background convergent substitutions [16]. Such a model has been proposed [16,17], but the current implementation can only work one branch at a time, not along a tree topology.

Second, although it is an important part of the model, the phenotype is here considered in an extremely naive fashion. In particular, we have made no effort to incorporate a distribution of fitness effects, whereby different sites would contribute differently to the phenotype under consideration, and therefore to the fitness [34]. Using such a distribution would be key to understanding why some sites, those of large effect, undergo convergent evolution while others, with smaller effects, do not. It could also indicate to users what effect sizes are large enough to be detected in a given experimental setting, and what effect sizes are just too small to be detected.

Third, several known confounding factors have not been simulated. In particular, we have not incorporated bGC in our simulations, and we have not incorporated population-level processes that would allow polymorphisms to cross speciation events (incomplete lineage sorting, ILS) and would increase the levels of polymorphisms present at the tips of the trees. We have not investigated several factors that are likely to affect the ability of the methods to detect convergent amino acid evolution such as tree size, tree shape and branch lengths (but see [18]). Our simulation pipeline can, however, be used to study such parameters.

With these caveats in mind, our simulations show that existing methods are much better at detecting convergent profile changes rather than convergent selection efficacy rescalings. Further, detection of convergent profile changes is improved when selection efficacy is high, possibly because this increases the frequency of type 1 substitutions. They also show that model-based methods, which explicitly rely on profiles, perform better than other methods.

Moving forward, we can think of three complementary directions for improving methods aiming to detect adaptive convergent evolution in amino acid sequences. In all cases, they will be based on profile methods anchored in a mechanistic modelling of sequence evolution. As a first direction, we need to complement models of sequence evolution so that, in addition to profile changes, we can also accurately detect changes in selection efficacy and distinguish those adaptive processes from confounding factors such as bGC and ILS. Further anchoring the model in population genetics theory may allow the interpreting of detected sites in terms of the fitness advantage they provide. As a second direction, we need to improve the computational efficiency of model-based inference. This should be a major concern here, because datasets are getting larger every year; algorithmic or mathematical developments will probably be necessary to fit such complex models onto large datasets. In this respect, one intriguing result of this study is the performance of the multinomial method. This simplistic method ignores nearly everything of the complexities of codon models of sequence evolution and yet achieves a performance that rivals them in some conditions. Correcting the multinomial method for phylogenetic inertia could provide even better performances, and it may be possible to improve it further while keeping its excellent speed. Finally, we have only tested the methods' ability to detect individual convergent sites; some methods (e.g. msd) can also employ a statistical procedure to detect convergent genes by combining site-wise evidence. Alternatively, TDG09 has a procedure to control its false positive rate, and diffsel estimates parameters based on entire alignments, not single sites. None of those features have been tested but are crucial for application to real data, in particular, for application to genome-wide datasets. Future analyses will have to investigate these aspects.

## Supplementary Material

Supplementary methods

## Data accessibility

Our pipeline's code is available at https://gitlab.in2p3.fr/pveber/reviewphiltrans. It contains everything required to reproduce our results. Detailed reproduction instructions are given in electronic supplementary material, §9. All intermediate data used to produce our results (approx. 20 Go) are available from the Dryad Digital Repository: https://doi.org/10.5061/dryad.57hr00q/1 [35].
