# Supplementary material for: Detecting adaptive convergent amino acid evolution
Source: Philos Trans R Soc Lond B Biol Sci. 2019 Jun 3;374(1777):20180234. doi: 10.1098/rstb.2018.0234 (PMC6560273; doi:10.1098/rstb.2018.0234)
Supplement: Supplementary methods [file rstb20180234supp1.docx]

Supplementary material for

Detecting adaptive convergent amino acid evolution

Carine Rey^*1^, Vincent Lanore^*2^, Philippe Veber^2^, Laurent Guéguen^2^, Nicolas Lartillot^2^, Marie Sémon^§1^, Bastien Boussau^§2^.

^*^: Those authors contributed equally

^§^: Those authors contributed equally

^1^: Univ Lyon, Université Claude Bernard Lyon 1, ENS de Lyon, CNRS UMR 5239, INSERM U1210, LBMC, F-69007, Lyon, France.

^2^: Univ Lyon, Université Claude Bernard Lyon 1, CNRS UMR 5558, LBBE, F-69100, Villeurbanne, France.

##

[1/ Distinction between foreground and background convergent substitutions](#_o94oyxkr160m) 2

[2/ Model of codon sequence evolution used in the simulations](#_9hho0kdjtdjd) 2

[3/ Tree topologies](#_j3nqlqy79skd) 3

[4/ Selection of amino acid profiles used in the simulations](#_6uvpaiq5ldqc) 3

[Step 1: Estimation of site-wise profiles based on mammalian alignments](#_quko458n4562) 4

[Step 2: Selection of a subset of non-redundant Bloom profiles](#_wywghshcg3i6) 4

[5/ Choice of NeS used in the simulations](#_2qc8nrnqvjt4) 6

[6/ Standardizing the output of detection methods](#_33knqh57narz) 8

[7/ Comparaison of the AUC values for the 4 phylogenies](#_zhnqg8z4hefl) 10

[8/ Impact of using separate alignments for diffsel](#_dkwudbm5vcl4) 10

[9/ How to reproduce our results](#_8lhmjfo2hvwm) 11

[10/ Ranks and alignments for previously identified sites in empirical data](#_5wthvrk5aiv) 13

[**References**](#_16qasntcmb2d) **16**

## 1/ Distinction between foreground and background convergent substitutions

##
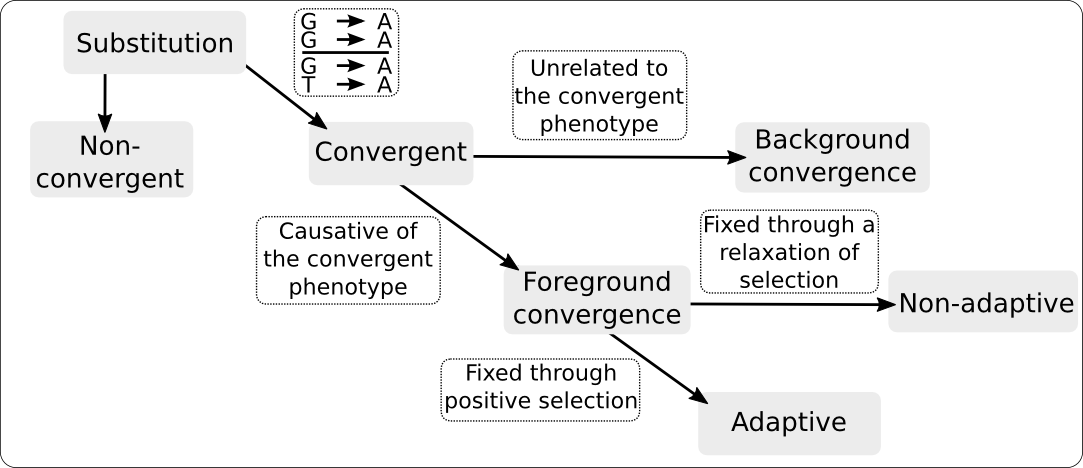


**Figure S1:** Overview of the distinction between foreground and background convergent substitutions.

## 2/ Model of codon sequence evolution used in the simulations

We performed simulations using bppseqgen [(1,2)](https://paperpile.com/c/lLSNnz/MLbX+OKt9) under a codon model in which all sites are considered independent of each other, without rate heterogeneity across sites. This codon model accounts for site-wise amino acid preferences and for variations in the efficacy of selection. For a given site, it has two levels, with level 1 modelling DNA mutations between codons, and level 2 modelling the fixation of these mutations, similar to [(3–5)](https://paperpile.com/c/lLSNnz/6nc9q+T6nEn+s4tCu).

At level 1, mutations between codons are based on a Jukes-Cantor model [(6)](https://paperpile.com/c/lLSNnz/3bZEv), allowing only one nucleotide substitution per codon per instantaneous time.

At level 2, fixation probabilities are dependent on two parameters: the vector of amino acid frequencies, and the efficacy of selection NeS. If the frequencies of two amino acids A and B are $f{}_{A}$ and $f{}_{B}$, the fixation probability of the mutation from A to B is proportional to $-NeS \frac{log(f{}_{A}/f{}_{B})}{1-(f{}_{A}/f{}_{B}){}^{NeS}}$as per Equation 2 in [(7)](https://paperpile.com/c/lLSNnz/KVbpq).

Finally, to fit with the usual modelling in phylogenetics, the resulting generator matrix is normalized so that there is one substitution per codon per unit of time at equilibrium.

## 3/ Tree topologies


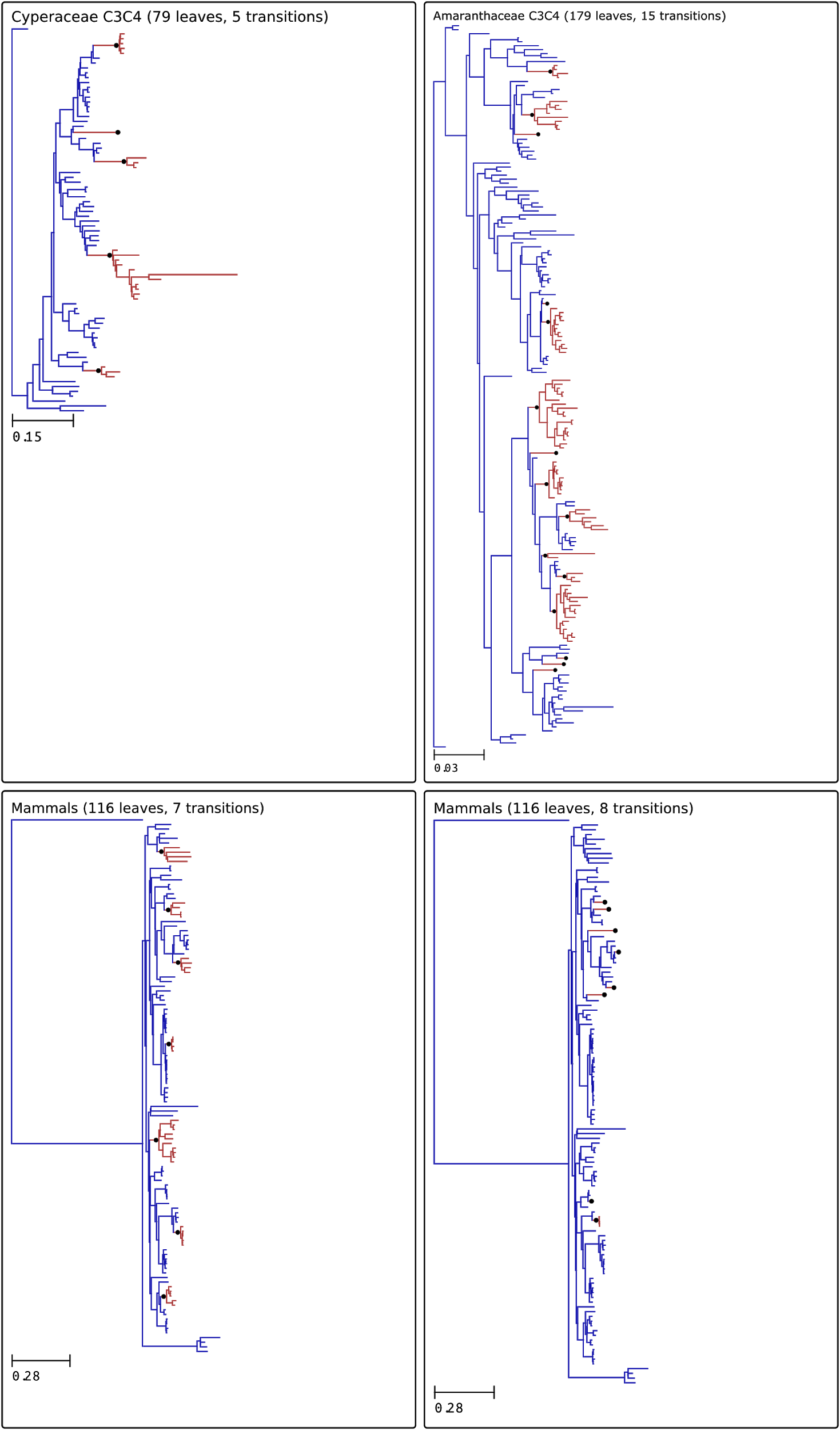


**Figure S2:** Overview of the 4 phylogenies. Convergent clades are in red. Black dots indicate branch with a convergent transition. Convergent transitions of the Cyperaceae [(8)](https://paperpile.com/c/lLSNnz/eN4sA) and the Amaranthaceae [(9)](https://paperpile.com/c/lLSNnz/VT1aS) phylogenies correspond to C3C4 transitions. For the mammals phylogenies [(10)](https://paperpile.com/c/lLSNnz/7d5NO), convergent transitions are placed on branches leading to xeric species for the 8 transitions tree. For the 7 transitions tree, convergent transitions have been placed unrelated to a specific phenotype, but homogeneously in the tree such as convergent clades are small subtrees. Tree topologies and convergent transition annotations are available at <https://gitlab.in2p3.fr/pveber/reviewphiltrans>.

## 4/ Selection of amino acid profiles used in the simulations

Amino acid profiles are vectors of frequencies for each of the 20 amino acids. These profiles are related to vectors of amino acid fitnesses [(4)](https://paperpile.com/c/lLSNnz/T6nEn). Several sets of amino acid profiles have been proposed in previous works. Our aim was to obtain a set of non-redundant and realistic amino acid profiles that would readily describe the frequencies of amino acids for a site of a protein alignment that has been subjected to a constant and homogeneous set of selective forces throughout its evolution. This criterion discards sets of profiles estimated from amino acid alignments [(11)](https://paperpile.com/c/lLSNnz/W4TE3), as those must contain sites that have undergone changing selective pressures during their history. We therefore relied on profiles that have been obtained during wet lab experiments involving mutagenesis and fitness measurements on viruses [(12)](https://paperpile.com/c/lLSNnz/4oMSz). We collected 1389 profiles by combining the profiles obtained in [(12)](https://paperpile.com/c/lLSNnz/4oMSz). However these profiles (named below “Bloom profiles”) are highly redundant (Figure S3-A), and therefore some filtering is necessary before using them to simulate changes in fitness profiles. We reasoned that a good subset of these profiles would be one that can fit most sites of empirical alignments well. To obtain this subset, we used a two step approach. First, we learned thousands of site-wise amino acid profiles from alignments of mammalian coding DNA sequences. Second, we estimated the set of Bloom profiles that best fits 95% of these sites. This 95% criterion is arbitrary but corresponds to our hypothesis that only a minority of sites have undergone a change in selective pressures during their evolution, and therefore could not be well fitted by a single Bloom profile.

### Step 1: Estimation of site-wise profiles based on mammalian alignments

Starting from Orthomam v6 [(13)](https://paperpile.com/c/lLSNnz/2Py8m), 100 CDS were randomly sampled, for 37 placental mammals. The alignments were trimmed for misaligned regions and short frameshifts, yielding a total of 64721 aligned coding sites. To achieve faster computation, these were further subdivided into 5 sets of 20 genes. For each set, the alignments were concatenated, resulting in 5 independent multiple sequence alignment of approximately 13000 coding positions each (gene lists and alignments available…). For each alignment, the mutation-selection model of Rodrigue et al [(5)](https://paperpile.com/c/lLSNnz/s4tCu), such as implemented in phylobayes mpi version 1.8 [(14)](https://paperpile.com/c/lLSNnz/daX0C) was run in 2 replicates, for approximately 4500 cycles. Counting a burnin of 500, these runs were used to compute the site-specific marginal posterior mean fitness profiles (64721 sites, 2 replicates).

### Step 2: Selection of a subset of non-redundant Bloom profiles

For each of the 64721 profiles, we identified the amino acid profile from Bloom’s data that maximizes the multinomial likelihood. Finally, we chose the smallest set of Bloom profiles that provides ML fits for 95% of the 64721 profiles. This set contains 263 Bloom profiles. Amino acid frequencies of each profile are available in the gitlab repository of the pipeline and presented in Figure S3-B.

A
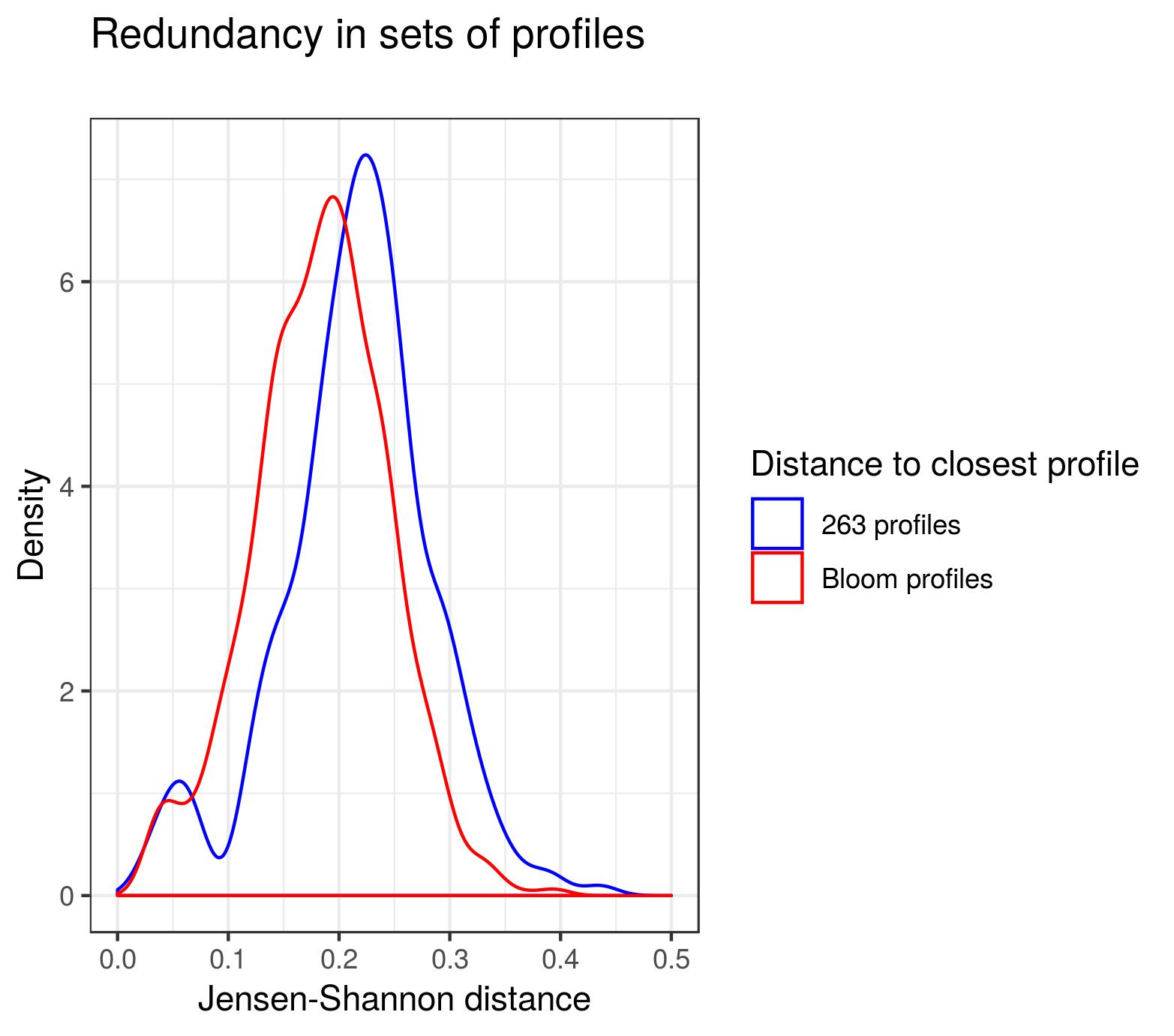


B


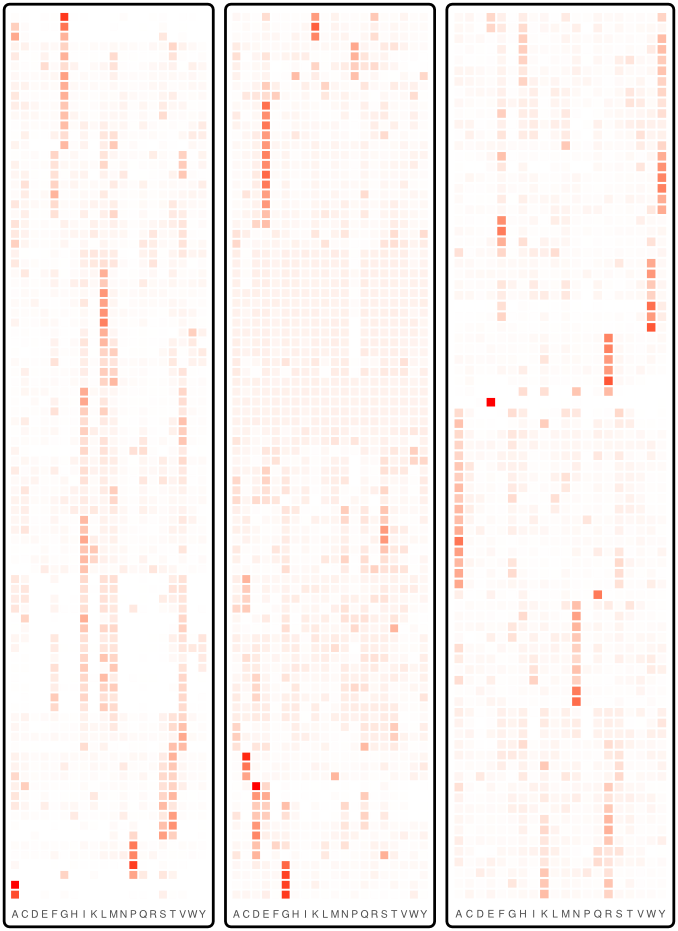


**Figure S3:** Amino acid profiles selected from the Bloom dataset. A. Redundancy in sets of profiles is higher in Bloom dataset than in the 263 retained profiles. Distribution of the distance to the closest profile, measured as the Jensen-Shannon distance. B. The 263 Bloom profiles retained after removing redundancy, ordered by Jensen-Shannon distance. Each row corresponds to a profile and each column to the frequency of an amino acid in this profile. Amino acid frequencies for each profile are available at <https://gitlab.in2p3.fr/pveber/reviewphiltrans>.

## 5/ Choice of NeS used in the simulations

The profiles we used were derived from assays in which the fitness of all 20 amino acids are estimated in competition experiments in the lab, resulting in fitness profiles. Those fitness profiles are not representative of fitnesses that would be obtained in the wild but are highly dependent upon the experimental conditions that were used. In particular, we expect that the scaled selection coefficient NeS would change the fitness (Figure 1). To estimate a realistic range of NeS values to use in our simulations, we computed descriptive statistics on empirical and simulated alignments. We reasoned that NeS values that generate simulated alignments with descriptive statistics similar to those of empirical alignments would be appropriate.

We computed several statistics:

- dS: the number of synonymous substitutions per site summed over the entire tree. Note here that dS is not defined as is typically done in codon models.
- dN: the number of non-synonymous substitutions per site summed over the entire tree. Note here that dN is not defined as is typically done in codon models.
- dN/dS: the ratio of the above two
- Normalized amino acid diversity: the total number of different amino acid states per site, divided by dS

Those statistics were computed by fitting bppml [(3)](https://paperpile.com/c/lLSNnz/6nc9q) on empirical or simulated codon alignments, and then mapping substitutions using mapnh [(3,15)](https://paperpile.com/c/lLSNnz/6nc9q+z1tkQ). The results were then analyzed using Python and R scripts.


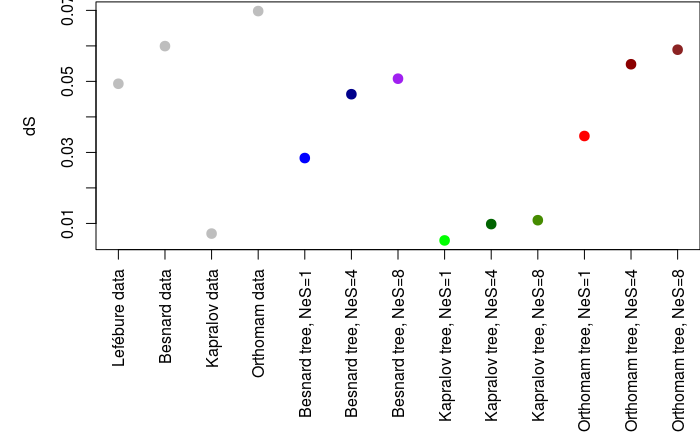


**Figure S4:** dS statistic computed on empirical (4 first columns) and simulated data. The simulated data are based on 3 different tree topologies obtained from the literature [(8–10,16)](https://paperpile.com/c/lLSNnz/0eOFp+eN4sA+VT1aS+7d5NO), using 3 different NeS values (1, 4, 8), under a homogeneous model and a single amino acid profile per site.


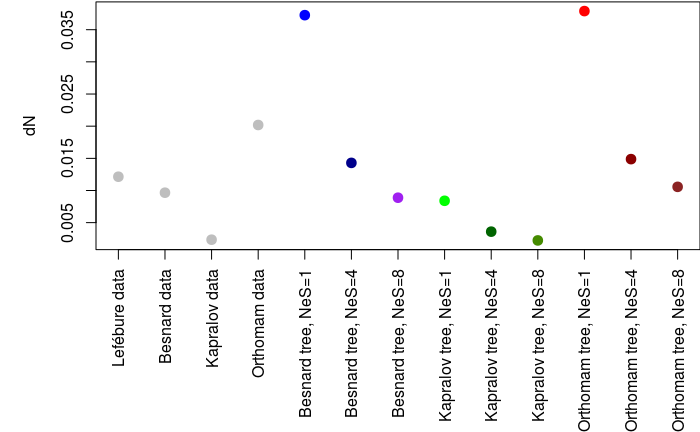


**Figure S5:** dN statistic computed on empirical (4 first columns) and simulated data. Legend is as above.


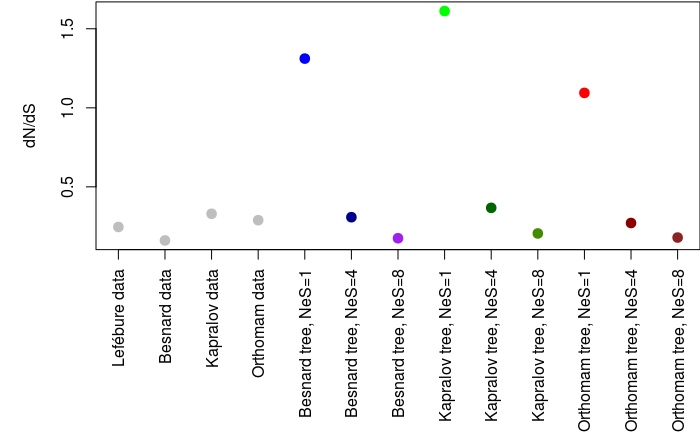


**Figure S6:** dN/dS statistic computed on empirical (4 first columns) and simulated data. Legend is as above.


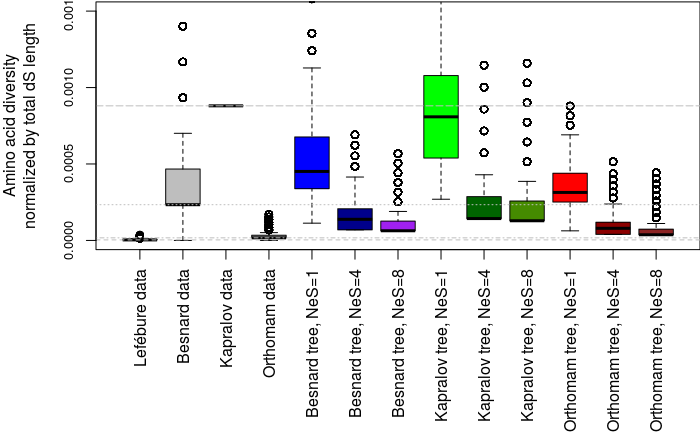


**Figure S7:** Normalized diversity statistic computed on empirical (4 first columns) and simulated data. Legend is as above.

Based on the figures above, we considered that the NeS values 4 and 8 in particular provided data sets that were representative of what is observed in nature.

## 6/ Standardizing the output of detection methods

In order to compare results across methods, it was necessary to standardize their output. We have decided to use a score between 0 and 1 for every site, where higher means the site is more likely to be convergent.

- For the **Identical** method, the score of a site corresponds to the number of substitutions to the most frequent amino acid found among species with the convergent phenotype. Amino acid frequencies are calculated using bppancestor from [(17)](https://paperpile.com/c/lLSNnz/CuoVu) using LG08 as substitution model.
- For the **Topological** method, the score of a site is the posterior probability between 2 models using the same parameters except the tree topology, where in the H0 hypothesis, the true topology is used and in the Ha hypothesis a convergent topology where all convergent leaves where group together as defined in [(18,19)](https://paperpile.com/c/lLSNnz/MEv1X+omyaG)) and implemented in [(20)](https://paperpile.com/c/lLSNnz/281Bi). The likelihood of each model is calculated by bppseqgen from [(17)](https://paperpile.com/c/lLSNnz/CuoVu) using LG08 as substitution model.
- For **TDG09**, the score of a site correspond to (one minus) the p-value of the likelihood ratio test calculated by the method. We can not use the FDR values given by the method because sites from the H0 and the Ha hypothesis are independently scored which is incompatible with the calculation of the FDR.
- The **diffsel** method, published in [(21)](https://paperpile.com/c/lLSNnz/PO96p), uses a codon-based differential-selection model to detect patterns of convergent evolution. A MCMC approach is used to compute posterior distributions of parameter values, from which statistics can be gathered to establish convergence scores. The original method looks at the probability p(i, aa) over the posterior distribution, for site i and amino acid aa, that the differential selection effect for aa is greater than the mean over all amino acids. If this probability is close to 0 or to 1 (i.e., above 1-c or below c, for some cutoff c<0.5), then it is considered that there is a statistically significant differential effect for this site and amino acid. In order to compare diffsel with other methods, we compute s(i, aa) = 2 * |0.5 - p(i, aa)| to get a score between 0 and 1 such that higher is better. We then compute diffsel_max(i) = max_aas_(s(i, aa)), the maximum score on all amino acids, to get a score per site. Another thing to note is that diffsel is not completely site-independent as it estimates branch lengths across sites. Running it on separate 100% Ha and 100% H0 alignements may thus give skewed results compared to a more realistic case with 2% Ha and 98% H0 in the same alignment. However, early experiments showed (see supplementary section 8), that in practice the difference was small. We thus decided to keep Ha and H0 alignements separate, as alignments with 98% H0 and 2000 Ha sites would have been prohibitively expensive to process (several months per alignment).
- For **PCOC**, the score of a site is the value of the PCOC model given by the method.
- For the **multinomial** method, the score of a site corresponds to 1 - p-value, using the p-value of a Chi2 test to compare the amino acid frequency vector for the extant species with the ancestral phenotype to that obtained for the extant species with the convergent phenotype.
- For **msd**, the score of a site corresponds to 1 - p-value given by the method.

## 7/ Comparaison of the AUC values for the 4 phylogenies


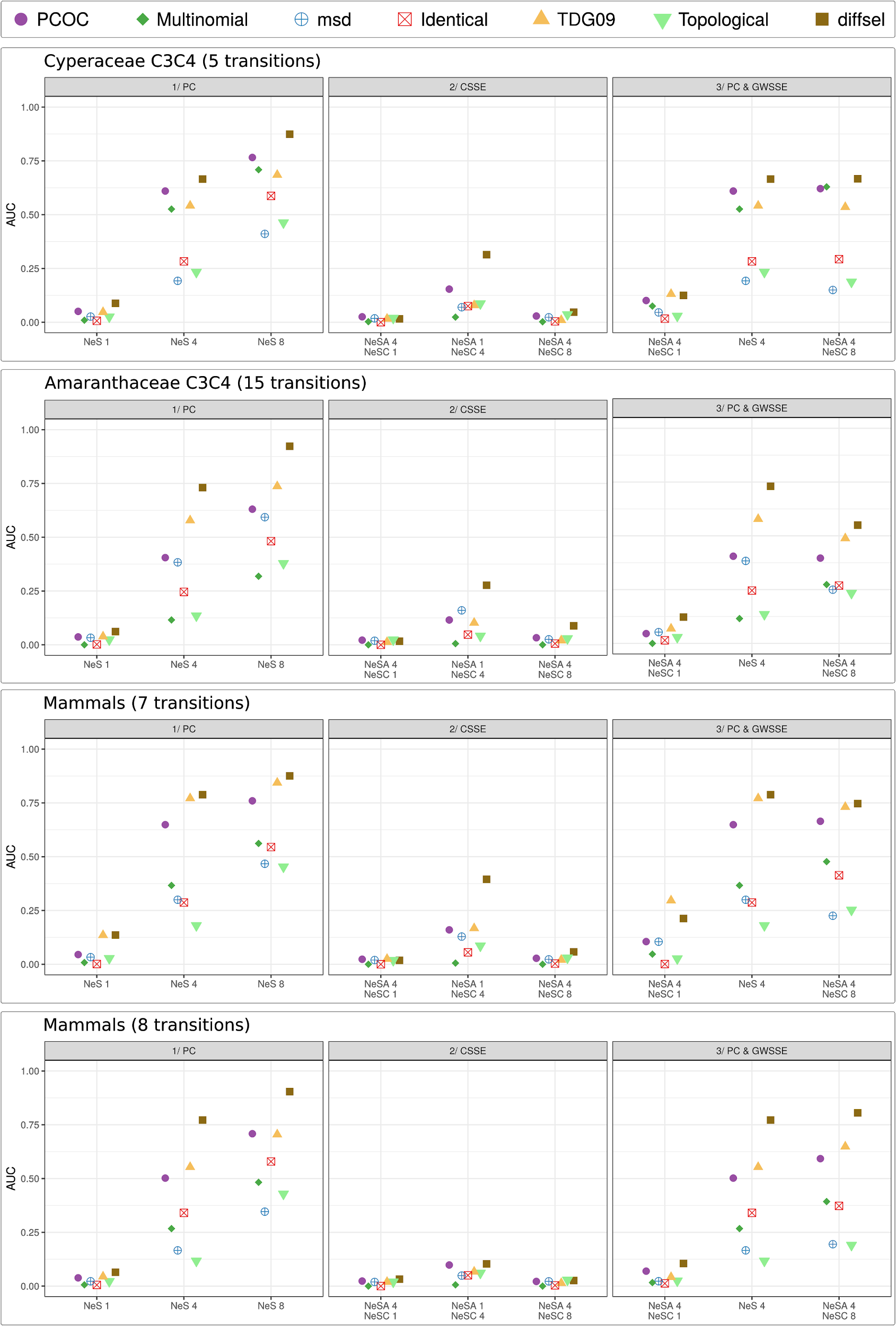


**Figure S8:** Comparison of the AUC values for the 4 phylogenies in the 3 kinds of adaptive convergent cases that have been simulated: 1) a convergent profile change in the selective pressure, 2) a convergent scaling of selection efficacy, and 3) a convergent profile change combined with a selection efficacy scaling. Cyperaceae row corresponds to results presented in Figure 4. The genome-wide selection efficacy (NeS**_A_**) remains the same in 1) and is changed to a convergent selection efficacy (NeS**_c_** ) in Ha 2) and Ha 3).

## 8/ Impact of using separate alignments for diffsel

Diffsel is the only method among the ones we considered that is not site-independent. Indeed, it estimates branch lengths across sites. Running diffsel on separate H0 and Ha alignments may lead to different branch lengths between alignments, making scores difficult to compare across alignments.

In order to estimate the impact of using separate alignments, we have conducted a small experiment outside of our main pipeline where we run diffsel on three different alignments:

- a 900-site H0 alignment simulated on the Cyperaceae C3C4 tree with NeS=4;
- a 100-site Ha alignment simulated with profile change and the same tree and NeS;
- the 1000-site concatenation of the two first alignments.

The concatenated alignment allows diffsel to estimate branch lengths across a 90/10 mix of Ha and H0 sites, without knowledge of which is which. This corresponds to a real alignment with a few convergent sites and a lot of non-convergent ones. We have considered in the paper that, in real data, the H0/Ha ratio would probably be closer to 98/2. Running diffsel on an alignment with such a H0/Ha ratio would, however, require a very large alignment in order to keep a reasonable number of Ha sites.

Figure S9 presents the results of these runs in the form of recall/precision plots corrected to mimic a 98/2 H0/Ha ratio, like in the paper. Table S1 gives figures for AUC and best recall at 90% precision. We see that the concatenated runs have slightly worse results in terms of AUC, but still stay ahead of PCOC, the second best method in the same conditions. Recall at 90% precision stays the same. Overall, assuming a similar behaviour across NeS hypotheses, using diffsel on concatenates would not change any of the qualitative results presented in the paper, but would have been extremely compute-intensive. Indeed, we would have needed to simulate 2000 Ha sites, which means 2000*98/2=98000 H0 sites so as to run Diffsel in conditions similar to the other methods. Such a run would take several months with the current (single-threaded) diffsel implementation.

**Table S1:** AUC and best recall at 90% precision for diffsel on 900/100 H0/Ha concat and non-concat simulations. AUC for pcoc in similar conditions (from Figure 3) is given for comparison.

|  | **Separate** | **Concat** |
| --- | --- | --- |
| **AUC diffsel_max** | 0.707 | 0.621 |
| **R90 diffsel_max** | 0.51 | 0.51 |
| **AUC pcoc** | 0.609 | |


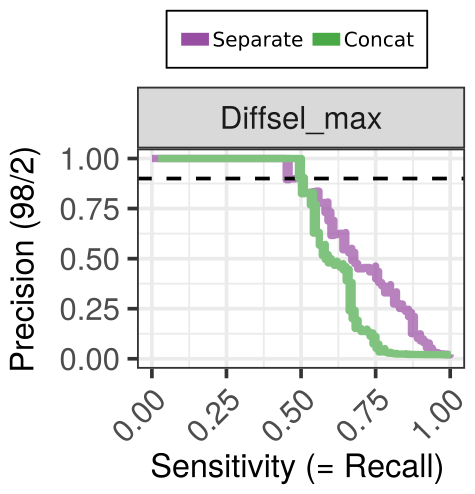


**Figure S9:** Precision/recall plots for diffsel on separate alignments (purple) or concatenated alignments (green).

The results presented in Figure S9 have been computed by running diffsel and scripts manually outside of the pipeline. Intermediate results and reproduction steps are available at <ftp://pbil.univ-lyon1.fr/pub/lanore/diffsel_concat_experiment.tar.gz> (300Mo) during the review process (and would be hosted on Dryad after acceptance).

## 9/ How to reproduce our results

Apart from the observed computing times for detection methods, results in this paper should be exactly reproducible.

**Computing resources:** keep in mind that the whole pipeline can take a very long time to run depending of the number of trees, the number of hypotheses and the number of simulated sites. Experiments presented in the paper took five days to run on a 24-core machine for 2000 sites, 4 trees and 12 hypotheses. Suitable computing resources should be used to reproduce our run (at least 24 cores for five days or 48 for two days and a half). Running all the experiments on a 4-core laptop would take approximately a month.

**Installed software:** a working version of docker is necessary to run our pipeline. Installation instructions for docker are beyond the scope of this reproduction guide. Be aware that docker requires root privileges and is thus unlikely to be available on traditional clusters. Virtual machines are an easy way to circumvent this problem.

Apart from docker, opam (an OCaml package manager) and a few OCaml packages are necessary to run the pipeline. The following commands should install all of that on a Debian-based linux distribution:

sudo apt install m4 opam zlib1g-dev

opam init

eval `opam config env`

opam switch 4.06.1

eval `opam config env`

opam pin add biocaml https://github.com/biocaml/biocaml.git

opam pin add bistro https://github.com/pveber/bistro.git#first-class-file-dumps -n

opam install ppx_csv_conv bistro ocamlify biocaml

All other software is packaged in docker images and thus requires no installation. The use of docker images guarantees that the various programs in the pipeline (scripts, detection methods, python and R packages…) are exactly identical to the ones we used in our experiments.

**Pipeline version:** the pipeline itself is available through the repository at <https://gitlab.in2p3.fr/pveber/reviewphiltrans>. The version that we used for the paper corresponds to tag “v1.01” (commit d3e25255c8315a29a81a9cc8a691005568dee57e).

**Build and run the pipeline:**

git clone https://gitlab.in2p3.fr/pveber/reviewphiltrans

cd reviewphiltrans

git checkout v1.01

make

make install

make analyses

**Basic troubleshooting:** an error at compilation (make) probably indicates that opam or some packages are not properly installed: try re-running the opam commands given above. An error at runtime mentioning docker probably indicates that docker is improperly installed or configured.

**Pipeline output:** if the pipeline successfully runs, a folder will be created called reviewphiltrans/example/outdir_analyses. This folder contains all intermediate (e.g., simulated data, method outputs, .tsv files used for plots…) and final (plots) results. Results are organized in sub-folders with explicit names. The plots used in the paper are called <tree-name>.recall_precision_ok.pdf.

**Paper intermediate results:** all the intermediate results of the runs used in the paper have been conserved to aid reproducibility. They are temporarily available at <ftp://pbil.univ-lyon1.fr/pub/lanore/outdir_full.tar.gz> (16Go) during the review process (and would be hosted on Dryad after acceptance). A lighter version (500Mo) of the intermediate results without the diffsel traces (which take 800Mo per run) is available at <ftp://pbil.univ-lyon1.fr/pub/lanore/outdir_without_chains.tar.gz>.

**Empirical data runs:** the run on empirical data used to make Figure 5, Figure S10 and Figure S11 was performed separately from the main run. The version of the pipeline used was commit 5598b5015371f6f80274544c808dbaeae1fe0c0f.

The “empircial data” version of the pipeline requires a folder with real trees and nucleotide alignments. For our run, it was structured like this:

real_data/

├── amaranth

│ ├── C4AmaranthaceaePolyroot.fna

│ └── C4AmaranthaceaePolyroot.nhx

└── besnard2009

├── cyp_coding.fna

└── cyp_coding.nhx

The command used was: reviewphiltrans realdata --indir real_data/ --outdir real_outdir --preview-mode --np 4 --diffsel --seed 20181206

Intermediate results for the empirical runs have been conserved and would be available on Dryad after acceptation.

## 10/ Ranks and alignments for previously identified sites in empirical data

Here we discuss in more details the results of the detection methods obtained on the two empirical data sets.

Profile methods tend to perform better than other methods. In particular, in both data sets, Tdg09 appears in excellent agreement with the original publications.

The Amaranthaceae data set appears more difficult for almost all methods than the Cyperaceae data set, perhaps because the Amaranthaceae data has fewer transitions than the Cypearaceae data set. However, msd performs better on the Amaranthaceae than on the Cyperaceae. In particular, in Amaranthaceae, the multinomial method gives the same score to all the sites and therefore does not detect any convergent evolution: for all sites it finds that a single vector of frequencies fits the data better than two vectors. The Topological and Identical methods do not perform very well on this data set either.

In the Amaranthaceae data, three sites are given high ranks (=low probabilities of being convergent) by diffsel. This may seem surprising because those sites had been found positive in the initial study based on a diffsel analysis. However, diffsel was then used with three conditions (ancestral branches, convergent branches, and their sister clades), whereas we only used 2. The difference is therefore due to this particular change in the options given to diffsel.


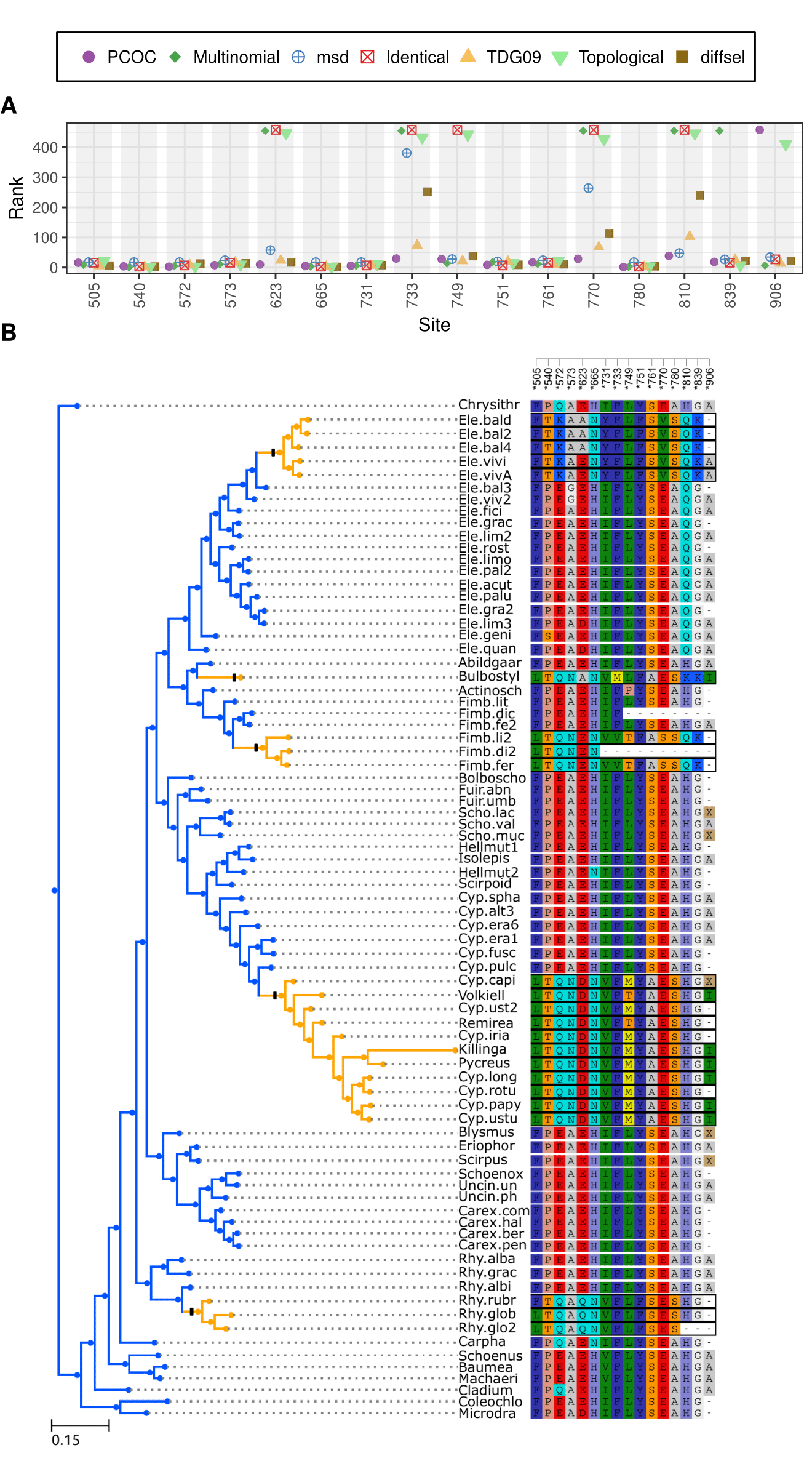


**Figure S10:** For all sites identified in the original paper, combined figures with rank per method (panel A) and alignment (panel B) for the Cyperaceae dataset [(8)](https://paperpile.com/c/lLSNnz/eN4sA).


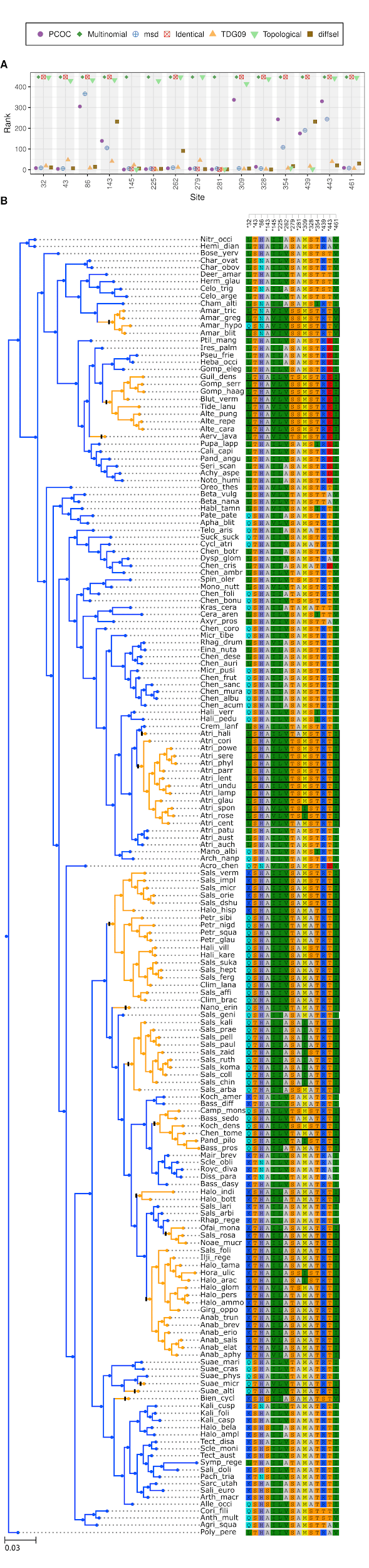


**Figure S11:** For all sites identified in the original paper, combined figures with rank per method (panel A) and alignment (panel B) for for the Amaranthaceae dataset [(9,21)](https://paperpile.com/c/lLSNnz/PO96p+VT1aS).

# References

1. [Guéguen L, Gaillard S, Boussau B, Gouy M, Groussin M, Rochette NC, et al. Bio++: efficient extensible libraries and tools for computational molecular evolution. Mol Biol Evol. 2013 Aug;30(8):1745–50.](http://paperpile.com/b/lLSNnz/MLbX)

2. [Rey C, Guéguen L, Sémon M, Boussau B. Accurate detection of convergent amino-acid evolution with PCOC. Mol Biol Evol [Internet]. 2018 Jul 7; Available from:](http://paperpile.com/b/lLSNnz/OKt9) <http://dx.doi.org/10.1093/molbev/msy114>

3. [Guéguen L, Gaillard S, Boussau B, Gouy M, Groussin M, Rochette NC, et al. Bio++: efficient extensible libraries and tools for computational molecular evolution. Mol Biol Evol. 2013 Aug;30(8):1745–50.](http://paperpile.com/b/lLSNnz/6nc9q)

4. [Halpern AL, Bruno WJ. Evolutionary distances for protein-coding sequences: modeling site-specific residue frequencies. Mol Biol Evol. 1998 Jul;15(7):910–7.](http://paperpile.com/b/lLSNnz/T6nEn)

5. [Rodrigue N, Philippe H, Lartillot N. Mutation-selection models of coding sequence evolution with site-heterogeneous amino acid fitness profiles. Proc Natl Acad Sci U S A. 2010 Mar 9;107(10):4629–34.](http://paperpile.com/b/lLSNnz/s4tCu)

6. [Jukes TH, Cantor CR. Evolution of Protein Molecules. In: Mammalian Protein Metabolism. 1969. p. 21–132.](http://paperpile.com/b/lLSNnz/3bZEv)

7. [Rodrigue N, Lartillot N. Detecting Adaptation in Protein-Coding Genes Using a Bayesian Site-Heterogeneous Mutation-Selection Codon Substitution Model. Mol Biol Evol. 2017 Jan;34(1):204–14.](http://paperpile.com/b/lLSNnz/KVbpq)

8. [Besnard G, Muasya AM, Russier F, Roalson EH, Salamin N, -A. Christin P. Phylogenomics of C4 Photosynthesis in Sedges (Cyperaceae): Multiple Appearances and Genetic Convergence. Mol Biol Evol. 2009;26(8):1909–19.](http://paperpile.com/b/lLSNnz/eN4sA)

9. [Kapralov MV, Smith JAC, Filatov DA. Rubisco Evolution in C4 Eudicots: An Analysis of Amaranthaceae Sensu Lato. PLoS One. 2012;7(12):e52974.](http://paperpile.com/b/lLSNnz/VT1aS)

10. [Douzery EJP, Scornavacca C, Romiguier J, Belkhir K, Galtier N, Delsuc F, et al. OrthoMaM v8: a database of orthologous exons and coding sequences for comparative genomics in mammals. Mol Biol Evol. 2014 Jul;31(7):1923–8.](http://paperpile.com/b/lLSNnz/7d5NO)

11. [Le SQ, Lartillot N, Gascuel O. Phylogenetic mixture models for proteins. Philos Trans R Soc Lond B Biol Sci. 2008 Dec 27;363(1512):3965–76.](http://paperpile.com/b/lLSNnz/W4TE3)

12. [Bloom JD. Identification of positive selection in genes is greatly improved by using experimentally informed site-specific models [Internet]. 2016. Available from:](http://paperpile.com/b/lLSNnz/4oMSz) <http://dx.doi.org/10.1101/037689>

13. [Ranwez V, Delsuc F, Ranwez S, Belkhir K, Tilak M-K, Douzery EJP. OrthoMaM: A database of orthologous genomic markers for placental mammal phylogenetics. BMC Evol Biol. 2007 Nov 30;7(1):241.](http://paperpile.com/b/lLSNnz/2Py8m)

14. [Rodrigue N, Lartillot N. Site-heterogeneous mutation-selection models within the PhyloBayes-MPI package. Bioinformatics. 2014 Apr 1;30(7):1020–1.](http://paperpile.com/b/lLSNnz/daX0C)

15. [Dutheil JY, Galtier N, Romiguier J, Douzery EJP, Ranwez V, Boussau B. Efficient selection of branch-specific models of sequence evolution. Mol Biol Evol. 2012 Jul;29(7):1861–74.](http://paperpile.com/b/lLSNnz/z1tkQ)

16. [Lefébure T, Morvan C, Malard F, François C, Konecny-Dupré L, Guéguen L, et al. Less effective selection leads to larger genomes. Genome Res. 2017 Jun;27(6):1016–28.](http://paperpile.com/b/lLSNnz/0eOFp)

17. [Guéguen L, Gaillard S, Boussau B, Gouy M, Groussin M, Rochette NC, et al. Bio++: efficient extensible libraries and tools for computational molecular evolution. Mol Biol Evol. 2013 Aug;30(8):1745–50.](http://paperpile.com/b/lLSNnz/CuoVu)

18. [Parker J, Tsagkogeorga G, Cotton JA, Liu Y, Provero P, Stupka E, et al. Genome-wide signatures of convergent evolution in echolocating mammals. Nature. 2013 Oct 10;502(7470):228–31.](http://paperpile.com/b/lLSNnz/MEv1X)

19. [Castoe TA, de Koning APJ, Kim H-M, Gu W, Noonan BP, Naylor G, et al. Evidence for an ancient adaptive episode of convergent molecular evolution. Proc Natl Acad Sci U S A. 2009 Jun 2;106(22):8986–91.](http://paperpile.com/b/lLSNnz/omyaG)

20. [Rey C, Guéguen L, Sémon M, Boussau B. Accurate detection of convergent amino-acid evolution with PCOC. Mol Biol Evol [Internet]. 2018 Jul 7; Available from:](http://paperpile.com/b/lLSNnz/281Bi) <http://dx.doi.org/10.1093/molbev/msy114>

21. [Parto S, Lartillot N. Correction: Molecular adaptation in Rubisco: Discriminating between convergent evolution and positive selection using mechanistic and classical codon models. PLoS One. 2018 Apr 18;13(4):e0196267.](http://paperpile.com/b/lLSNnz/PO96p)
